# Atrophic Dermatofibroma: A Case Report With Dermoscopic and Histopathological Correlation

**DOI:** 10.7759/cureus.81342

**Published:** 2025-03-28

**Authors:** Oday A Alzaidieen, Ebrahim M Ebrahim, Waad Kadori, Muna Abuhejleh

**Affiliations:** 1 Dermatology, Primary Health Care Corporation, Doha, QAT; 2 Medical Education, Hamad Medical Corporation, Doha, QAT; 3 Pathology, Hamad General Hospital, Doha, QAT

**Keywords:** atrophic dermatofibroma, benign fibrous histiocytoma, benign skin lesions, dermatofibroma, dermatofibrosarcoma protuberans

## Abstract

Atrophic dermatofibroma (ADF) is a rare variant of dermatofibroma (DF). In this article, we report a case of an asymptomatic 44-year-old woman with a concerning atrophic skin lesion on her leg. Clinical examination and dermoscopy were not enough to confirm the diagnosis, so surgical excision with a histopathological report led to the final diagnosis of atrophic dermatofibroma and the reassurance of the patient. Atrophic dermatofibroma should be considered in the clinician’s differential diagnosis of atrophic lesions. Despite its benign nature, more sinister pathologies must be ruled out.

## Introduction

Dermatofibroma (DF), also known as benign fibrous histiocytoma, is a common benign skin lesion arising from proliferating fibroblasts and histiocytes within the dermis [1]. DF typically presents as a firm papule or nodule on the legs of young or middle-aged adults. Eruptive dermatofibromas have been observed in patients with autoimmune disorders (e.g., lupus erythematosus) and atopic dermatitis and in the setting of immunosuppression (e.g., HIV infection) [2]. An uncommon variant, atrophic dermatofibroma (ADF), presents as a depressed or atrophic lesion, potentially leading to clinical misdiagnosis [3].

Histologically, DFs show dermal proliferation of spindled fibrohistiocytic cells forming intersecting fascicles with collagen entrapment [4]. There are several histological variants of DFs, including the palisading variant, which features nuclear palisading resembling Verocay bodies, and the keloidal variant, which contains keloidal collagen [4]. These variants may lead to diagnostic confusion with other neoplasms or conditions [4]. The etiology of DF remains unclear, with hypotheses suggesting either a neoplastic process or a reactive response to minor skin trauma [5]. On the other hand, ADF shows a similar proliferation of fibrohistiocytic cells but within a thinned dermis, with a notable loss of elastic fibers, as demonstrated by Elastica van Gieson staining [6,7]. This loss of elastic fibers may be due to elastophagocytosis, where the tumor cells phagocytize the elastic fibers [7].

Unless they cause symptoms or cosmetic concerns, DFs from all variants are generally benign and do not require treatment. In such cases, surgical excision may be performed. Lastly, it is important to differentiate DFs from other skin lesions, such as basal cell carcinomas, dermal nevi, and dermatofibrosarcoma protuberans, which can have similar clinical presentations but require different management strategies [8]. Clinicians should consider ADF in the differential diagnosis of atrophic skin lesions to avoid misdiagnosis and ensure appropriate management [3,6]. In this article, we report a case of an atrophic skin lesion with a definitive diagnosis of ADF.

## Case presentation

A 44-year-old woman presented to our outpatient dermatology clinic with an asymptomatic skin lesion on her right lower leg. She had noticed the lesion for several years without any change in size or color. It appeared spontaneously, with no history of preceding trauma, injections, or insect bites. Her past medical history is unremarkable except for hypertension. Systemic examination was unremarkable, with no palpable lymphadenopathy in the lower limbs or groin.

**Figure 1:**
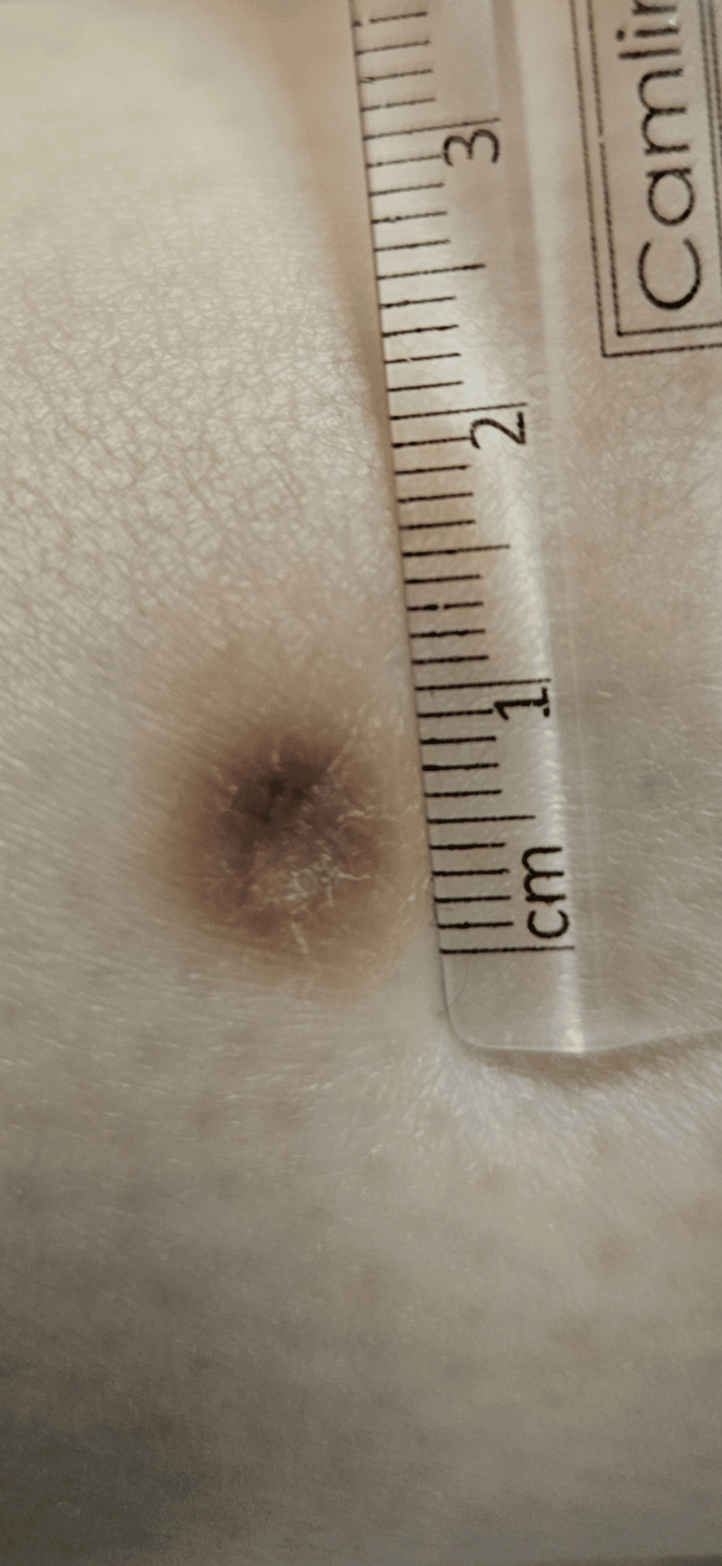
A solitary 8 mm depressed firm hyperpigmented macule on the right leg.

**Figure 2:**
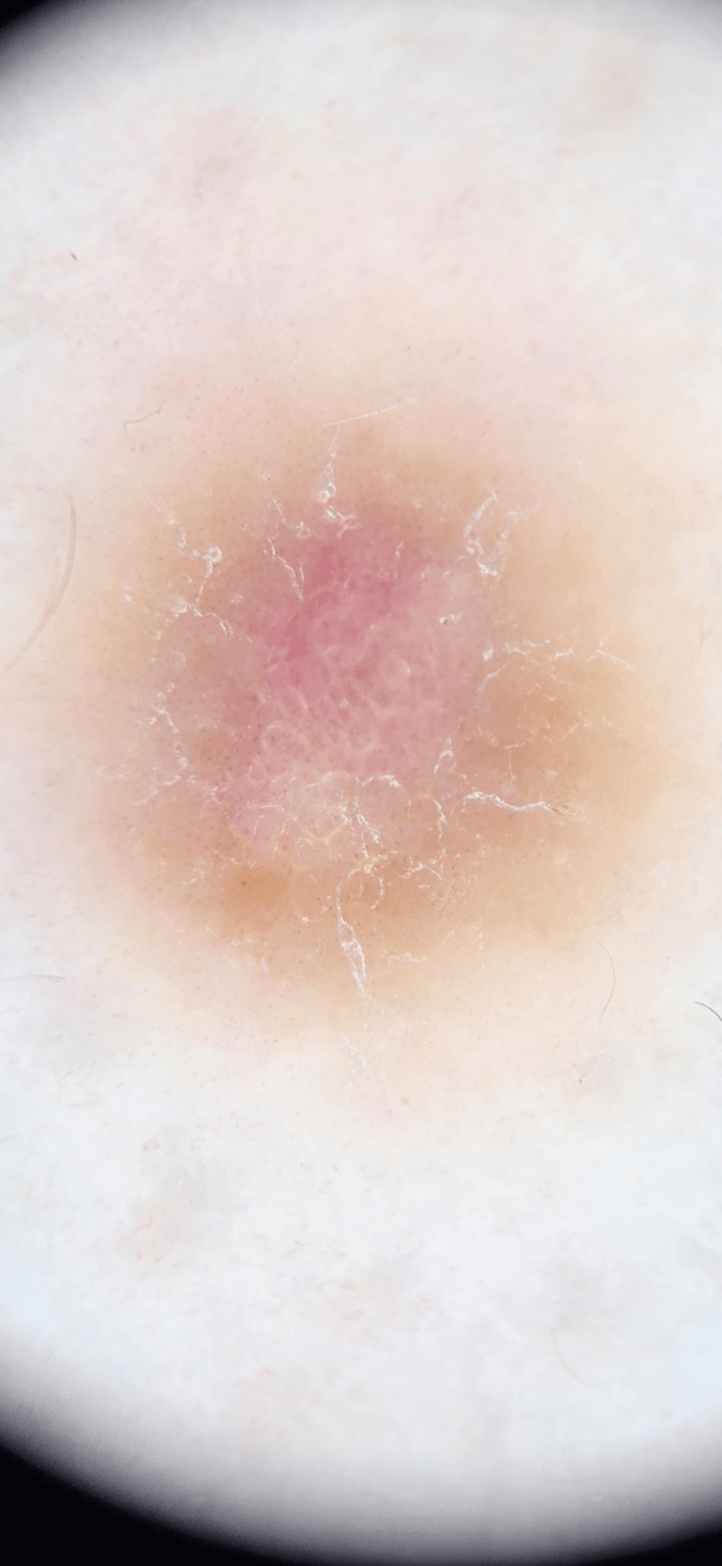
Dermoscopy: pink and white scar-like center with pigmented network peripherally.

Dermatologic examination revealed an 8 mm hyperpigmented macule with an atrophic center (Figure 1). The lesion exhibited a firm texture upon bilateral compression. Dermoscopy showed a central pink and white scar-like area with a pigmented network at the periphery, which cannot confirm a diagnosis of DF (see Figure 2).

In view of the clinical and dermoscopic findings, a 3 cm elliptical excision with 1 mm margins was performed under local anesthesia. Two layers of sutures were used to close the defect, followed by regular dressing. Stitches were removed after two weeks without any complications. Reassurance was given to the patient about the benign diagnosis of this skin lesion, and there is no risk of recurrence after excision.

**Figure 3:**
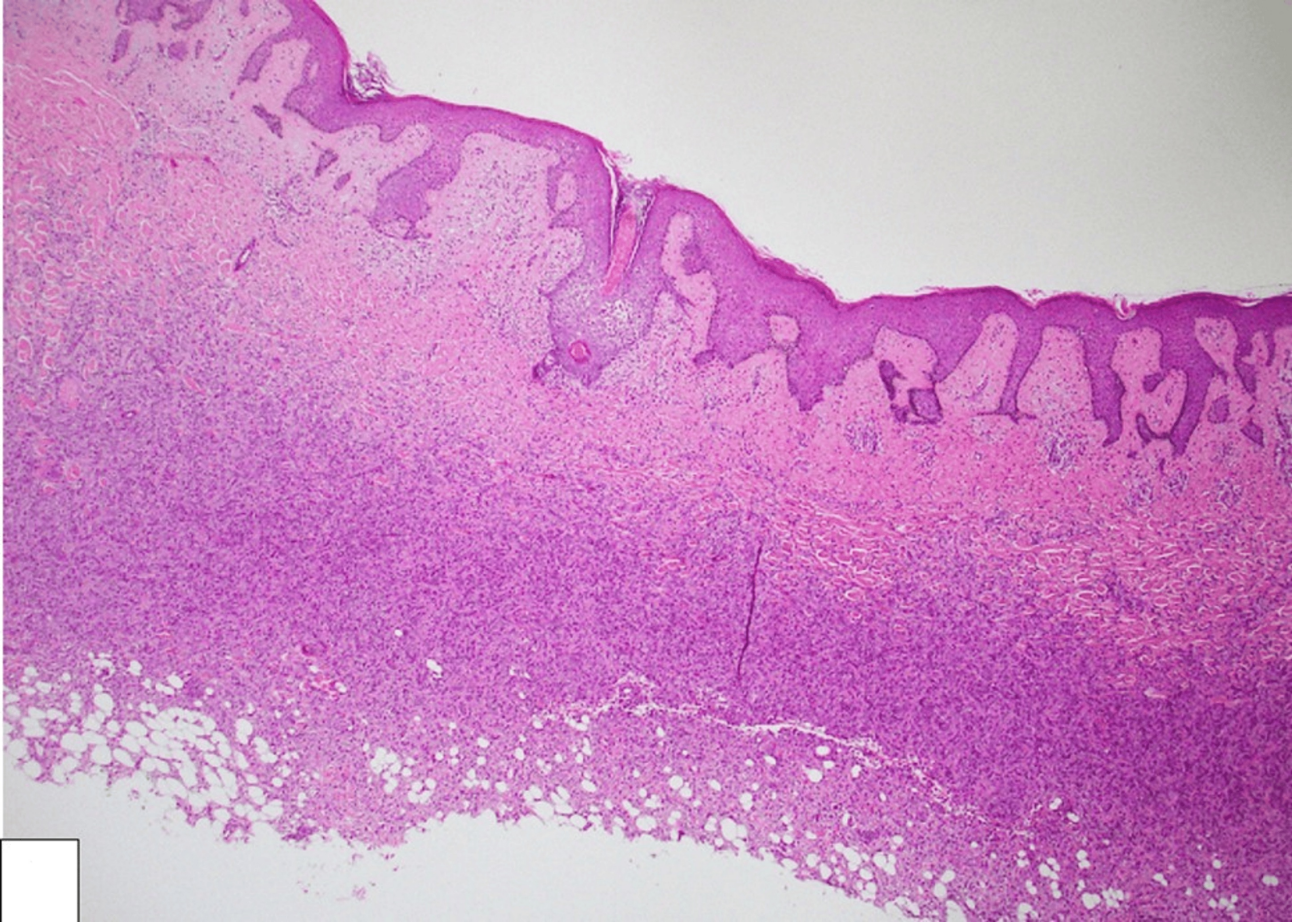
Histological image showing a cellular dermal-based lesion, composed of bland spindle cells (H&E: ×100).

**Figure 4:**
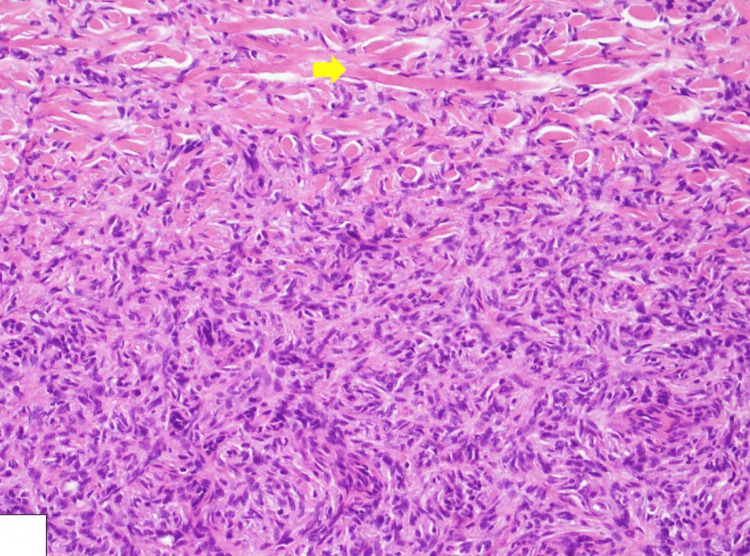
Histological image showing a spindle cell lesion with entrapped thick collagen bundles at the periphery (yellow arrow) (H&E: ×400).

Histopathological examination revealed a complete excision without lesion on surgical margins, a cellular dermal-based lesion, composed of bland spindle cells with elongated nuclei and eosinophilic cytoplasm, arranged in a storiform pattern (see Figure 3). Additionally, entrapped thick collagen bundles at the periphery were noted with extension to the subcutaneous tissue (Figure 4). No cytological atypia or mitotic activity was identified, featuring DF. Further immunohistochemical analysis showed positive staining for cluster of differentiation 68 (CD68) and factor XIIIa, confirming the diagnosis of DF, while negative staining for cluster of differentiation 34 (CD34) ruled out atrophic dermatofibrosarcoma protuberans.

## Discussion

DF is one of the most common benign tumors of the skin and typically presents as asymptomatic firm skin color or hyperpigmented papule or nodule on the limbs, usually with a history of preceding minor trauma or insect bite, and examination shows a characteristic “dimple sign” when lateral pressure is applied because of deep attachment to subcutaneous tissue. However, several atypical subtypes, including atrophic, giant, hyperkeratotic, and ulcerated, have been identified in uncommon locations such as the scalp, upper back, and face [9]. ADF, one of the atypical clinical forms, despite its rarity, was reported to account for approximately 2% of all DFs [10].

A definitive diagnosis of ADF requires excisional biopsy and histopathological examination, as it can be mistaken for basal cell carcinoma, atrophic dermatofibrosarcoma protuberans, atrophoderma, morphea, or anetoderma [9].

Dermoscopic examinations can aid the diagnosis of DF; however, some cases may lack the typical features, such as a central white scar-like area surrounded by a pigmented network [11].

The histopathological examination of entrapped collagen bundles is the hallmark findings of all types of DF [12]. In the immunohistochemical examination, positive staining for factor XIIIa and negative staining for CD34 were particularly important in distinguishing ADF from atrophic dermatofibrosarcoma protuberans. Subcutaneous tissue, as well as the dermis, is also involved in atrophic dermatofibrosarcoma [13].

## Conclusions

While surgical excision is the mainstay of treatment for dermatofibroma with a low recurrence rate, the cosmetic result of a scar after surgical excision is worse than the dermatofibroma appearance itself. ADF is a rare, atypical variant that presents as a depressed skin lesion, resembling basal cell carcinoma or atrophic dermatofibrosarcoma protuberans. Dermoscopic examination alone is insufficient to exclude malignancy; therefore, excision and histopathological analysis are necessary. This case of ADF demonstrated atypical dermoscopic features but typical histopathological findings.
